# Supplementary material for: Avoidable Mortality between Metropolitan and Non-Metropolitan Areas in Korea from 1995 to 2019: A Descriptive Study of Implications for the National Healthcare Policy
Source: Int J Environ Res Public Health. 2022 Mar 15;19(6):3475. doi: 10.3390/ijerph19063475 (PMC8955663; doi:10.3390/ijerph19063475)
Supplement: Supplementary file 1 [file ijerph-19-03475-s001.zip › ijerph-1555766-supplementary.pdf]

Supplementary Table S1. Age standardized all-cause mortality rates per 100,000 persons (95% confidence interval) of metropolitan and non-metropolitan areas by sex from 1995 to 2019

Year	Total			Male			Female		
	All-area	Metropolitan	Non-metropolitan	All-area	Metropolitan	Non-metropolitan	All-area	Metropolitan	Non-metropolitan
1995	733.1 (680.0 - 786.1)	654.2 (604.1 - 704.3)	786.2 (731.2 - 841.2)	1020.8 (958.2 - 1083.4)	878.9 (820.8 - 937.0)	1115.8 (1050.3 - 1181.3)	526.0 (481.1 - 571.0)	492.8 (449.3 - 536.4)	547.6 (501.8 - 593.5)
1996	704.7 (652.6 - 756.7)	635.4 (586.0 - 684.8)	752.6 (698.8 - 806.4)	980.7 (919.3 - 1042.1)	856.9 (799.5 - 914.3)	1065.6 (1001.6 - 1129.5)	504.6 (460.6 - 548.6)	475.3 (432.6 - 518.1)	524.5 (479.7 - 569.4)
1997	704.2 (652.2 - 756.2)	632.7 (583.4 - 682.0)	753.9 (700.1 - 807.7)	972.1 (911.0 - 1033.2)	844.0 (787.1 - 900.9)	1060.2 (996.4 - 1124.0)	508.7 (464.5 - 552.9)	477.8 (435.0 - 520.7)	529.8 (484.7 - 574.9)
1998	683.1 (631.9 - 734.4)	620.9 (572.1 - 669.8)	727.3 (674.5 - 780.2)	943.4 (883.2 - 1003.6)	829.6 (773.2 - 886.1)	1023.0 (960.3 - 1085.7)	492.4 (448.9 - 535.9)	467.1 (424.8 - 509.5)	510.4 (466.2 - 554.7)
1999	665.7 (615.1 - 716.3)	600.9 (552.8 - 648.9)	711.2 (658.9 - 763.5)	911.5 (852.3 - 970.7)	798.8 (743.4 - 854.2)	990.4 (928.7 - 1052.1)	484.3 (441.2 - 527.5)	453.3 (411.6 - 495.1)	505.5 (461.4 - 549.5)
2000	646.1 (596.3 - 696.0)	591.3 (543.6 - 638.9)	685.4 (634.1 - 736.7)	881.0 (822.8 - 939.2)	778.5 (723.8 - 833.2)	953.3 (892.7 - 1013.8)	471.7 (429.1 - 514.2)	448.9 (407.4 - 490.4)	487.9 (444.6 - 531.2)
2001	611.5 (563.0 - 659.9)	561.5 (515.1 - 608.0)	647.8 (597.9 - 697.7)	834.8 (778.2 - 891.4)	744.6 (691.2 - 798.1)	899.7 (840.9 - 958.5)	445.0 (403.6 - 486.3)	422.6 (382.3 - 462.9)	461.1 (419.0 - 503.2)
2002	597.3 (549.4 - 645.2)	549.2 (503.3 - 595.1)	632.8 (583.5 - 682.1)	810.0 (754.2 - 865.8)	722.1 (669.4 - 774.8)	874.0 (816.0 - 931.9)	438.4 (397.4 - 479.5)	416.5 (376.5 - 456.5)	454.6 (412.8 - 496.4)
2003	569.3 (522.6 - 616.1)	519.3 (474.7 - 564.0)	606.4 (558.1 - 654.7)	773.4 (718.9 - 827.9)	687.2 (635.8 - 738.5)	837.0 (780.3 - 893.7)	415.5 (375.5 - 455.4)	390.0 (351.3 - 428.7)	434.0 (393.1 - 474.8)
2004	546.6 (500.8 - 592.5)	503.0 (459.0 - 547.0)	579.6 (532.4 - 626.8)	746.4 (692.8 - 799.9)	667.7 (617.1 - 718.4)	805.4 (749.7 - 861.0)	395.8 (356.8 - 434.8)	376.0 (338.0 - 414.0)	410.7 (371.0 - 450.4)
2005	524.0 (479.2 - 568.9)	485.4 (442.2 - 528.5)	553.4 (507.3 - 599.5)	714.2 (661.8 - 766.5)	647.9 (598.0 - 697.8)	764.6 (710.4 - 818.8)	381.7 (343.4 - 420.0)	361.6 (324.3 - 398.8)	396.3 (357.3 - 435.4)
2006	499.4 (455.6 - 543.2)	462.3 (420.1 - 504.4)	527.9 (482.9 - 572.9)	683.6 (632.4 - 734.9)	617.8 (569.1 - 666.5)	733.8 (680.7 - 786.9)	361.1 (323.9 - 398.4)	342.7 (306.5 - 379.0)	374.9 (336.9 - 412.8)
2007	481.9 (438.9 - 525.0)	445.3 (403.9 - 486.6)	510.6 (466.3 - 554.8)	659.4 (609.1 - 709.7)	597.3 (549.4 - 645.2)	707.8 (655.6 - 759.9)	349.6 (313.0 - 386.3)	330.6 (294.9 - 366.2)	364.0 (326.6 - 401.4)
2008	458.6 (416.6 - 500.6)	419.2 (379.0 - 459.3)	489.3 (445.9 - 532.6)	634.5 (585.1 - 683.8)	567.0 (520.3 - 613.7)	687.1 (635.7 - 738.4)	327.7 (292.3 - 363.2)	307.5 (273.2 - 341.9)	342.7 (306.4 - 379.0)
2009	438.8 (397.7 - 479.9)	403.3 (364.0 - 442.7)	466.9 (424.6 - 509.3)	606.4 (558.2 - 654.7)	547.4 (501.5 - 593.2)	653.1 (603.1 - 703.2)	312.5 (277.9 - 347.2)	293.0 (259.4 - 326.5)	327.6 (292.1 - 363.0)
2010	432.1 (391.4 - 472.8)	399.9 (360.7 - 439.1)	457.8 (415.9 - 499.7)	598.6 (550.7 - 646.6)	541.9 (496.3 - 587.5)	643.8 (594.0 - 693.5)	305.8 (271.5 - 340.1)	290.0 (256.6 - 323.3)	317.9 (282.9 - 352.8)
2011	415.6 (375.6 - 455.5)	385.5 (347.0 - 424.0)	440.1 (398.9 - 481.2)	575.7 (528.7 - 622.8)	522.7 (477.9 - 567.5)	618.5 (569.7 - 667.2)	293.3 (259.7 - 326.8)	278.5 (245.8 - 311.2)	305.1 (270.9 - 339.4)
2012	409.6 (369.9 - 449.3)	380.7 (342.5 - 419.0)	433.3 (392.5 - 474.1)	564.9 (518.3 - 611.5)	515.4 (470.9 - 559.8)	605.1 (556.9 - 653.3)	290.2 (256.8 - 323.6)	275.0 (242.5 - 307.5)	302.4 (268.3 - 336.5)
2013	388.8 (350.1 - 427.4)	364.1 (326.7 - 401.5)	409.3 (369.6 - 448.9)	533.3 (488.0 - 578.5)	488.2 (444.9 - 531.5)	570.0 (523.2 - 616.8)	276.3 (243.7 - 308.9)	264.8 (233.0 - 296.7)	285.8 (252.7 - 318.9)
2014	371.9 (334.1 - 409.7)	348.5 (311.9 - 385.1)	391.8 (353.0 - 430.6)	509.4 (465.1 - 553.6)	466.4 (424.1 - 508.7)	544.9 (499.1 - 590.6)	263.8 (232.0 - 295.7)	253.1 (222.0 - 284.3)	273.0 (240.6 - 305.4)
2015	363.6 (326.2 - 401.0)	337.8 (301.8 - 373.9)	385.1 (346.7 - 423.6)	494.5 (450.9 - 538.1)	450.4 (408.8 - 492.0)	530.9 (485.8 - 576.1)	259.7 (228.1 - 291.2)	246.0 (215.3 - 276.8)	270.8 (238.6 - 303.1)
2016	352.4 (315.6 - 389.2)	330.0 (294.4 - 365.7)	371.5 (333.7 - 409.2)	476.9 (434.1 - 519.7)	436.4 (395.5 - 477.4)	510.9 (466.6 - 555.3)	252.6 (221.4 - 283.7)	242.6 (212.1 - 273.1)	260.9 (229.3 - 292.6)
2017	339.2 (303.1 - 375.3)	314.8 (280.1 - 349.6)	359.9 (322.7 - 397.1)	457.3 (415.4 - 499.2)	417.4 (377.3 - 457.4)	491.1 (447.6 - 534.5)	243.2 (212.6 - 273.7)	229.7 (200.0 - 259.4)	254.1 (222.9 - 285.4)
2018	337.3 (301.3 - 373.3)	314.5 (279.8 - 349.3)	356.9 (319.9 - 393.9)	452.9 (411.2 - 494.6)	416.1 (376.1 - 456.1)	484.4 (441.3 - 527.5)	242.5 (212.0 - 273.1)	229.6 (199.9 - 259.3)	253.4 (222.2 - 284.6)
2019	319.6 (284.5 - 354.6)	296.9 (263.1 - 330.6)	339.3 (303.2 - 375.4)	429.6 (389.0 - 470.2)	393.8 (354.9 - 432.7)	460.7 (418.7 - 502.8)	228.7 (199.1 - 258.4)	215.6 (186.9 - 244.4)	239.6 (209.3 - 269.9)

Supplementary Table S2. Age standardized avoidable mortality rates per 100,000 persons (95% confidence interval) of metropolitan and non-metropolitan areas by sex from 1995 to 2019

Year	Total			Male			Female		
	All-area	Metropolitan	Non-metropolitan	All-area	Metropolitan	Non-metropolitan	All-area	Metropolitan	Non-metropolitan
1995	350.5 (313.8 - 387.2)	308.9 (274.4 - 343.3)	381.5 (343.2 - 419.8)	525.4 (480.5 - 570.4)	449.0 (407.5 - 490.5)	581.7 (534.5 - 629.0)	204.9 (176.8 - 232.9)	193.4 (166.1 - 220.7)	213.8 (185.2 - 242.5)
1996	331.6 (295.9 - 367.3)	290.7 (257.3 - 324.1)	362.5 (325.2 - 399.8)	497.1 (453.4 - 540.8)	423.5 (383.2 - 463.8)	551.7 (505.6 - 597.7)	193.2 (166.0 - 220.5)	180.0 (153.7 - 206.3)	203.8 (175.8 - 231.8)
1997	327.3 (291.8 - 362.7)	287.6 (254.4 - 320.8)	357.5 (320.4 - 394.6)	487.7 (444.5 - 531.0)	415.0 (375.1 - 455.0)	542.2 (496.5 - 587.8)	192.5 (165.3 - 219.7)	180.6 (154.3 - 206.9)	202.1 (174.3 - 230.0)
1998	311.2 (276.6 - 345.7)	274.6 (242.1 - 307.1)	339.4 (303.3 - 375.5)	466.4 (424.0 - 508.7)	398.7 (359.5 - 437.8)	517.5 (472.9 - 562.1)	180.3 (154.0 - 206.6)	169.6 (144.0 - 195.1)	189.2 (162.2 - 216.2)
1999	300.8 (266.8 - 334.7)	265.9 (233.9 - 297.8)	327.9 (292.4 - 363.4)	448.6 (407.1 - 490.1)	386.7 (348.2 - 425.3)	496.0 (452.4 - 539.7)	176.1 (150.1 - 202.1)	163.6 (138.5 - 188.7)	186.0 (159.2 - 212.7)
2000	295.0 (261.3 - 328.6)	264.3 (232.4 - 296.1)	319.3 (284.3 - 354.3)	438.4 (397.4 - 479.5)	381.3 (343.0 - 419.6)	482.7 (439.6 - 525.8)	173.4 (147.6 - 199.2)	163.9 (138.8 - 189.0)	181.5 (155.1 - 207.9)
2001	282.6 (249.7 - 315.6)	253.6 (222.4 - 284.9)	305.8 (271.5 - 340.0)	421.5 (381.3 - 461.8)	369.3 (331.7 - 407.0)	462.5 (420.3 - 504.6)	164.8 (139.6 - 190.0)	153.9 (129.6 - 178.2)	173.8 (148.0 - 199.6)
2002	278.0 (245.3 - 310.7)	248.2 (217.4 - 279.1)	301.9 (267.8 - 336.0)	412.1 (372.3 - 451.9)	358.8 (321.7 - 396.0)	454.3 (412.5 - 496.0)	163.4 (138.3 - 188.4)	152.0 (127.8 - 176.1)	172.7 (146.9 - 198.4)
2003	268.1 (236.0 - 300.2)	239.1 (208.7 - 269.4)	291.8 (258.3 - 325.3)	396.9 (357.8 - 435.9)	345.9 (309.5 - 382.4)	437.9 (396.9 - 479.0)	156.5 (132.0 - 181.1)	144.8 (121.2 - 168.4)	166.3 (141.0 - 191.6)
2004	253.8 (222.6 - 285.1)	227.6 (198.0 - 257.2)	275.5 (243.0 - 308.0)	377.7 (339.6 - 415.8)	330.2 (294.5 - 365.8)	416.3 (376.3 - 456.3)	146.1 (122.4 - 169.7)	136.7 (113.7 - 159.6)	154.1 (129.8 - 178.5)
2005	237.8 (207.5 - 268.0)	215.3 (186.5 - 244.0)	256.5 (225.2 - 287.9)	351.6 (314.8 - 388.3)	311.2 (276.7 - 345.8)	384.8 (346.3 - 423.2)	138.4 (115.3 - 161.4)	129.7 (107.4 - 152.1)	145.6 (121.9 - 169.2)
2006	222.0 (192.8 - 251.2)	200.0 (172.2 - 227.7)	240.5 (210.1 - 270.9)	329.8 (294.2 - 365.4)	291.2 (257.8 - 324.7)	361.8 (324.6 - 399.1)	126.9 (104.8 - 149.0)	117.9 (96.6 - 139.2)	134.4 (111.7 - 157.2)
2007	211.6 (183.0 - 240.1)	188.1 (161.2 - 214.9)	231.6 (201.7 - 261.4)	310.4 (275.9 - 344.9)	269.4 (237.2 - 301.6)	344.9 (308.5 - 381.3)	123.4 (101.7 - 145.2)	114.2 (93.3 - 135.2)	131.5 (109.0 - 153.9)
2008	199.3 (171.6 - 227.0)	176.0 (150.0 - 202.0)	219.2 (190.2 - 248.2)	293.0 (259.4 - 326.5)	253.0 (221.8 - 284.2)	326.9 (291.5 - 362.3)	115.2 (94.2 - 136.2)	105.8 (85.6 - 125.9)	123.2 (101.4 - 144.9)
2009	191.3 (164.2 - 218.5)	170.2 (144.6 - 195.8)	209.7 (181.3 - 238.1)	280.5 (247.7 - 313.4)	245.3 (214.6 - 275.9)	310.9 (276.3 - 345.4)	110.4 (89.8 - 131.0)	101.1 (81.4 - 120.8)	118.6 (97.2 - 139.9)
2010	184.3 (157.7 - 210.9)	165.5 (140.3 - 190.7)	200.8 (173.1 - 228.6)	270.4 (238.1 - 302.6)	238.1 (207.9 - 268.4)	298.5 (264.6 - 332.4)	105.6 (85.5 - 125.7)	98.2 (78.8 - 117.6)	112.1 (91.3 - 132.8)
2011	174.5 (148.7 - 200.4)	156.4 (131.9 - 180.9)	190.7 (163.6 - 217.7)	256.7 (225.3 - 288.1)	225.6 (196.2 - 255.1)	283.9 (250.9 - 316.9)	99.1 (79.5 - 118.6)	91.9 (73.1 - 110.7)	105.5 (85.4 - 125.6)
2012	165.8 (140.5 - 191.0)	149.2 (125.3 - 173.2)	180.6 (154.3 - 206.9)	244.3 (213.6 - 274.9)	216.8 (188.0 - 245.7)	268.5 (236.4 - 300.7)	93.0 (74.1 - 111.9)	85.9 (67.8 - 104.1)	99.5 (80.0 - 119.1)
2013	157.2 (132.6 - 181.8)	143.6 (120.1 - 167.0)	169.6 (144.0 - 195.1)	230.1 (200.3 - 259.8)	207.2 (179.0 - 235.4)	250.4 (219.4 - 281.4)	89.2 (70.7 - 107.7)	83.6 (65.7 - 101.5)	94.5 (75.4 - 113.5)
2014	150.0 (126.0 - 174.0)	136.4 (113.5 - 159.3)	162.5 (137.5 - 187.5)	219.3 (190.3 - 248.3)	197.2 (169.7 - 224.7)	239.3 (208.9 - 269.6)	84.9 (66.8 - 102.9)	78.9 (61.5 - 96.4)	90.5 (71.9 - 109.2)
2015	143.2 (119.7 - 166.6)	129.4 (107.1 - 151.7)	155.8 (131.3 - 180.2)	208.5 (180.2 - 236.8)	186.7 (159.9 - 213.4)	228.2 (198.6 - 257.8)	81.5 (63.8 - 99.2)	75.2 (58.2 - 92.2)	87.4 (69.1 - 105.7)
2016	136.9 (114.0 - 159.9)	124.3 (102.5 - 146.2)	148.6 (124.7 - 172.5)	199.2 (171.5 - 226.8)	179.0 (152.8 - 205.3)	217.6 (188.7 - 246.5)	78.0 (60.7 - 95.3)	72.7 (56.0 - 89.4)	82.9 (65.1 - 100.8)
2017	128.3 (106.1 - 150.5)	116.7 (95.5 - 137.8)	139.2 (116.1 - 162.4)	187.6 (160.7 - 214.4)	169.7 (144.2 - 195.2)	204.1 (176.1 - 232.1)	71.7 (55.1 - 88.3)	66.4 (50.4 - 82.4)	76.7 (59.5 - 93.9)
2018	124.9 (103.0 - 146.8)	113.4 (92.5 - 134.2)	136.0 (113.1 - 158.8)	181.6 (155.2 - 208.0)	164.6 (139.4 - 189.7)	197.6 (170.1 - 225.2)	70.4 (54.0 - 86.9)	64.7 (49.0 - 80.5)	76.0 (58.9 - 93.1)
2019	120.2 (98.7 - 141.7)	109.6 (89.1 - 130.1)	130.4 (108.0 - 152.8)	173.4 (147.6 - 199.2)	157.3 (132.7 - 181.9)	188.7 (161.8 - 215.6)	68.8 (52.6 - 85.1)	64.2 (48.5 - 79.9)	73.5 (56.7 - 90.2)

Supplementary Table S3. Age standardized preventable mortality rates per 100,000 persons (95% confidence interval) of metropolitan and non-metropolitan areas by sex from 1995 to 2019

Year	Total			Male			Female		
	All-area	Metropolitan	Non-metropolitan	All-area	Metropolitan	Non-metropolitan	All-area	Metropolitan	Non-metropolitan
1995	258.0 (226.6 - 289.5)	219.8 (190.8 - 248.9)	286.3 (253.1 - 319.5)	407.2 (367.7 - 446.8)	337.7 (301.6 - 373.7)	458.3 (416.3 - 500.2)	131.9 (109.4 - 154.4)	120.7 (99.2 - 142.3)	140.1 (116.9 - 163.3)
1996	246.0 (215.3 - 276.8)	209.3 (181.0 - 237.7)	273.6 (241.2 - 306.0)	388.9 (350.3 - 427.6)	324.0 (288.7 - 359.3)	437.4 (396.4 - 478.4)	124.6 (102.7 - 146.4)	112.2 (91.4 - 132.9)	134.0 (111.3 - 156.7)
1997	240.4 (210.0 - 270.8)	205.0 (176.9 - 233.0)	267.2 (235.1 - 299.2)	377.6 (339.5 - 415.7)	314.4 (279.6 - 349.1)	425.0 (384.6 - 465.4)	123.4 (101.6 - 145.2)	111.6 (90.9 - 132.3)	132.4 (109.8 - 155.0)
1998	225.0 (195.6 - 254.4)	191.9 (164.8 - 219.1)	250.2 (219.2 - 281.2)	355.1 (318.2 - 392.0)	295.3 (261.6 - 329.0)	400.1 (360.9 - 439.3)	113.7 (92.8 - 134.7)	103.1 (83.2 - 123.0)	122.1 (100.5 - 143.8)
1999	215.8 (187.0 - 244.6)	184.7 (158.1 - 211.4)	239.7 (209.4 - 270.0)	340.0 (303.8 - 376.1)	285.1 (252.0 - 318.2)	381.8 (343.5 - 420.1)	109.6 (89.1 - 130.1)	98.4 (79.0 - 117.8)	118.2 (96.9 - 139.5)
2000	210.0 (181.6 - 238.4)	182.4 (156.0 - 208.9)	231.5 (201.7 - 261.3)	330.7 (295.0 - 366.3)	280.8 (247.9 - 313.6)	369.1 (331.5 - 406.8)	106.6 (86.4 - 126.9)	97.2 (77.9 - 116.5)	114.1 (93.1 - 135.0)
2001	200.6 (172.8 - 228.4)	174.4 (148.5 - 200.2)	221.3 (192.1 - 250.4)	316.3 (281.5 - 351.2)	269.3 (237.2 - 301.5)	352.9 (316.1 - 389.7)	101.3 (81.5 - 121.0)	91.4 (72.6 - 110.1)	109.2 (88.7 - 129.7)
2002	198.6 (171.0 - 226.3)	171.6 (145.9 - 197.2)	220.0 (190.9 - 249.1)	312.2 (277.6 - 346.9)	264.0 (232.1 - 295.8)	350.1 (313.4 - 386.8)	100.6 (80.9 - 120.2)	90.2 (71.6 - 108.8)	108.7 (88.3 - 129.2)
2003	194.5 (167.1 - 221.8)	167.8 (142.4 - 193.2)	215.9 (187.1 - 244.7)	304.1 (269.9 - 338.3)	257.8 (226.3 - 289.2)	341.1 (304.9 - 377.3)	98.7 (79.2 - 118.1)	87.6 (69.3 - 106.0)	107.5 (87.2 - 127.9)
2004	184.1 (157.5 - 210.7)	160.3 (135.5 - 185.1)	203.4 (175.4 - 231.4)	290.1 (256.7 - 323.5)	246.8 (216.0 - 277.6)	324.9 (289.6 - 360.2)	91.0 (72.3 - 109.7)	82.7 (64.9 - 100.6)	97.8 (78.5 - 117.2)
2005	173.4 (147.6 - 199.2)	153.6 (129.3 - 177.9)	189.7 (162.7 - 216.7)	270.8 (238.5 - 303.0)	234.8 (204.8 - 264.9)	300.1 (266.1 - 334.0)	87.6 (69.3 - 105.9)	80.4 (62.8 - 98.0)	93.5 (74.5 - 112.4)
2006	160.9 (136.1 - 185.8)	141.5 (118.2 - 164.8)	177.1 (151.0 - 203.2)	252.8 (221.6 - 283.9)	218.3 (189.4 - 247.3)	281.3 (248.4 - 314.1)	79.3 (61.9 - 96.8)	71.8 (55.2 - 88.5)	85.5 (67.4 - 103.6)
2007	153.9 (129.6 - 178.2)	133.6 (111.0 - 156.3)	171.0 (145.4 - 196.6)	238.2 (208.0 - 268.5)	202.5 (174.6 - 230.4)	268.2 (236.1 - 300.3)	78.1 (60.8 - 95.5)	70.7 (54.2 - 87.2)	84.5 (66.5 - 102.5)
2008	145.9 (122.2 - 169.5)	126.4 (104.4 - 148.5)	162.4 (137.4 - 187.4)	225.9 (196.4 - 255.3)	192.1 (164.9 - 219.2)	254.6 (223.3 - 285.8)	73.4 (56.6 - 90.2)	66.1 (50.1 - 82.0)	79.5 (62.0 - 96.9)
2009	141.7 (118.4 - 165.1)	124.1 (102.3 - 146.0)	157.0 (132.4 - 181.6)	218.5 (189.5 - 247.5)	188.7 (161.8 - 215.6)	244.1 (213.5 - 274.8)	71.5 (54.9 - 88.1)	64.3 (48.6 - 80.0)	77.8 (60.5 - 95.1)
2010	135.9 (113.0 - 158.7)	120.1 (98.6 - 141.5)	149.8 (125.8 - 173.8)	209.8 (181.4 - 238.2)	182.4 (155.9 - 208.9)	233.7 (203.8 - 263.7)	67.8 (51.7 - 84.0)	61.9 (46.5 - 77.3)	73.1 (56.3 - 89.8)
2011	128.5 (106.3 - 150.7)	113.5 (92.6 - 134.4)	141.8 (118.5 - 165.2)	199.0 (171.4 - 226.7)	173.4 (147.6 - 199.2)	221.6 (192.4 - 250.7)	63.1 (47.6 - 78.7)	57.4 (42.6 - 72.3)	68.3 (52.1 - 84.5)
2012	120.8 (99.3 - 142.4)	107.5 (87.2 - 127.9)	132.8 (110.2 - 155.4)	187.6 (160.7 - 214.4)	164.6 (139.4 - 189.7)	207.9 (179.7 - 236.2)	58.5 (43.5 - 73.5)	53.8 (39.4 - 68.1)	63.0 (47.4 - 78.5)
2013	114.8 (93.8 - 135.7)	103.5 (83.6 - 123.5)	125.0 (103.1 - 146.9)	177.5 (151.3 - 203.6)	158.3 (133.6 - 182.9)	194.6 (167.2 - 221.9)	55.8 (41.1 - 70.4)	51.7 (37.6 - 65.7)	59.7 (44.5 - 74.8)
2014	108.8 (88.4 - 129.3)	97.8 (78.4 - 117.2)	119.0 (97.6 - 140.4)	168.3 (142.9 - 193.7)	150.0 (126.0 - 174.0)	184.9 (158.3 - 211.6)	52.6 (38.4 - 66.8)	48.3 (34.6 - 61.9)	56.7 (42.0 - 71.5)
2015	103.0 (83.1 - 122.9)	92.0 (73.2 - 110.8)	113.1 (92.3 - 134.0)	158.6 (133.9 - 183.3)	140.7 (117.4 - 163.9)	174.8 (148.9 - 200.7)	50.2 (36.3 - 64.1)	45.7 (32.4 - 58.9)	54.5 (40.0 - 68.9)
2016	97.9 (78.5 - 117.3)	87.5 (69.2 - 105.8)	107.5 (87.2 - 127.9)	150.8 (126.7 - 174.8)	133.7 (111.1 - 156.4)	166.4 (141.1 - 191.7)	47.4 (33.9 - 60.9)	43.5 (30.6 - 56.4)	51.1 (37.1 - 65.1)
2017	91.4 (72.7 - 110.1)	82.7 (64.8 - 100.5)	99.6 (80.1 - 119.2)	141.4 (118.0 - 164.7)	127.4 (105.3 - 149.5)	154.3 (130.0 - 178.7)	43.3 (30.4 - 56.2)	40.0 (27.6 - 52.4)	46.5 (33.1 - 59.9)
2018	88.8 (70.3 - 107.3)	79.5 (62.0 - 96.9)	97.8 (78.5 - 117.2)	136.3 (113.4 - 159.1)	122.2 (100.6 - 143.9)	149.6 (125.6 - 173.5)	42.9 (30.1 - 55.8)	38.6 (26.4 - 50.8)	47.2 (33.7 - 60.7)
2019	85.6 (67.5 - 103.8)	77.1 (59.9 - 94.4)	93.9 (74.9 - 112.9)	130.4 (108.0 - 152.8)	116.9 (95.7 - 138.1)	143.2 (119.7 - 166.7)	42.2 (29.5 - 55.0)	39.1 (26.8 - 51.3)	45.4 (32.2 - 58.6)

Supplementary Table S4. Age standardized treatable mortality rates per 100,000 persons (95% confidence interval) of metropolitan and non-metropolitan areas by sex from 1995 to 2019

Year	Total			Male			Female		
	All-area	Metropolitan	Non-metropolitan	All-area	Metropolitan	Non-metropolitan	All-area	Metropolitan	Non-metropolitan
1995	92.4 (73.6 - 111.3)	89.0 (70.5 - 107.5)	95.2 (76.1 - 114.4)	118.2 (96.9 - 139.5)	111.3 (90.6 - 132.0)	123.5 (101.7 - 145.3)	73.0 (56.3 - 89.8)	72.7 (56.0 - 89.4)	73.7 (56.9 - 90.5)
1996	85.6 (67.5 - 103.8)	81.4 (63.7 - 99.0)	88.9 (70.4 - 107.3)	108.2 (87.8 - 128.6)	99.5 (79.9 - 119.0)	114.2 (93.3 - 135.2)	68.7 (52.4 - 84.9)	67.8 (51.7 - 84.0)	69.8 (53.4 - 86.2)
1997	86.9 (68.6 - 105.2)	82.6 (64.8 - 100.4)	90.3 (71.7 - 108.9)	110.2 (89.6 - 130.7)	100.7 (81.0 - 120.3)	117.1 (95.9 - 138.4)	69.2 (52.9 - 85.5)	69.0 (52.7 - 85.3)	69.7 (53.4 - 86.1)
1998	86.2 (68.0 - 104.4)	82.7 (64.8 - 100.5)	89.2 (70.7 - 107.7)	111.3 (90.6 - 131.9)	103.4 (83.4 - 123.3)	117.3 (96.1 - 138.6)	66.5 (50.5 - 82.5)	66.4 (50.4 - 82.4)	67.1 (51.0 - 83.1)
1999	85.0 (66.9 – 103.0)	81.1 (63.5 - 98.8)	88.2 (69.8 - 106.6)	108.7 (88.2 - 129.1)	101.6 (81.8 - 121.4)	114.2 (93.3 - 135.2)	66.5 (50.5 - 82.5)	65.2 (49.4 - 81.0)	67.7 (51.6 - 83.9)
2000	85.0 (66.9 – 103.0)	81.8 (64.1 - 99.6)	87.8 (69.5 - 106.2)	107.8 (87.4 - 128.1)	100.5 (80.9 - 120.2)	113.6 (92.7 - 134.5)	66.8 (50.8 - 82.8)	66.7 (50.7 - 82.7)	67.4 (51.4 - 83.5)
2001	82.0 (64.3 - 99.8)	79.3 (61.8 - 96.7)	84.5 (66.5 - 102.5)	105.2 (85.1 - 125.3)	100.0 (80.4 - 119.6)	109.6 (89.1 - 130.1)	63.5 (47.9 - 79.1)	62.5 (47.0 - 78.0)	64.6 (48.9 - 80.4)
2002	79.4 (61.9 - 96.8)	76.7 (59.5 - 93.8)	81.9 (64.2 - 99.7)	99.8 (80.2 - 119.4)	94.8 (75.8 - 113.9)	104.2 (84.2 - 124.2)	62.8 (47.3 - 78.3)	61.8 (46.4 - 77.2)	63.9 (48.3 - 79.6)
2003	73.6 (56.8 - 90.4)	71.2 (54.7 - 87.8)	75.9 (58.8 - 93.0)	92.7 (73.9 - 111.6)	88.2 (69.8 - 106.6)	96.8 (77.5 - 116.1)	57.9 (43.0 - 72.8)	57.1 (42.3 - 72.0)	58.8 (43.7 - 73.8)
2004	69.7 (53.4 - 86.1)	67.4 (51.3 - 83.4)	72.1 (55.5 - 88.8)	87.5 (69.2 - 105.9)	83.4 (65.5 - 101.3)	91.4 (72.6 - 110.1)	55.1 (40.5 - 69.6)	53.9 (39.5 - 68.3)	56.3 (41.6 - 71.0)
2005	64.3 (48.6 - 80.1)	61.7 (46.3 - 77.1)	66.8 (50.8 - 82.9)	80.8 (63.2 - 98.4)	76.4 (59.3 - 93.5)	84.7 (66.7 - 102.7)	50.8 (36.8 - 64.7)	49.3 (35.6 - 63.1)	52.1 (38.0 - 66.3)
2006	61.1 (45.7 - 76.4)	58.5 (43.5 - 73.5)	63.4 (47.8 - 79.0)	77.0 (59.8 - 94.2)	72.9 (56.2 - 89.7)	80.6 (63.0 - 98.2)	47.6 (34.1 - 61.1)	46.0 (32.7 - 59.3)	49.0 (35.2 - 62.7)
2007	57.7 (42.8 - 72.6)	54.4 (40.0 - 68.9)	60.5 (45.3 - 75.8)	72.2 (55.5 - 88.8)	66.9 (50.9 - 83.0)	76.7 (59.5 - 93.8)	45.3 (32.1 - 58.5)	43.5 (30.6 - 56.5)	46.9 (33.5 - 60.4)
2008	53.4 (39.1 - 67.8)	49.6 (35.8 - 63.4)	56.8 (42.0 - 71.6)	67.1 (51.0 - 83.1)	60.9 (45.6 - 76.2)	72.3 (55.7 - 89.0)	41.8 (29.1 - 54.5)	39.7 (27.4 - 52.1)	43.7 (30.7 - 56.7)
2009	49.6 (35.8 - 63.4)	46.1 (32.8 - 59.4)	52.7 (38.5 - 66.9)	62.0 (46.6 - 77.5)	56.6 (41.8 - 71.3)	66.7 (50.7 - 82.7)	38.9 (26.7 - 51.1)	36.8 (24.9 - 48.7)	40.8 (28.2 - 53.3)
2010	48.4 (34.8 - 62.0)	45.4 (32.2 - 58.6)	51.0 (37.0 - 65.0)	60.6 (45.3 - 75.8)	55.7 (41.1 - 70.4)	64.8 (49.0 - 80.6)	37.8 (25.7 - 49.8)	36.3 (24.5 - 48.1)	39.0 (26.8 - 51.3)
2011	46.0 (32.7 - 59.3)	42.8 (30.0 - 55.7)	48.8 (35.1 - 62.5)	57.7 (42.8 - 72.6)	52.2 (38.1 - 66.4)	62.3 (46.9 - 77.8)	35.9 (24.2 - 47.7)	34.5 (23.0 - 46.0)	37.2 (25.2 - 49.1)
2012	44.9 (31.8 - 58.1)	41.7 (29.0 - 54.3)	47.8 (34.2 - 61.3)	56.7 (42.0 - 71.5)	52.2 (38.1 - 66.4)	60.6 (45.4 - 75.9)	34.5 (23.0 - 46.0)	32.2 (21.0 - 43.3)	36.6 (24.7 - 48.4)
2013	42.4 (29.7 - 55.2)	40.0 (27.6 - 52.4)	44.6 (31.5 - 57.7)	52.6 (38.4 - 66.8)	48.9 (35.2 - 62.7)	55.8 (41.2 - 70.4)	33.4 (22.1 - 44.8)	32.0 (20.9 - 43.1)	34.8 (23.2 - 46.4)
2014	41.1 (28.6 - 53.7)	38.6 (26.4 - 50.7)	43.5 (30.5 - 56.4)	51.0 (37.0 - 65.0)	47.2 (33.7 - 60.7)	54.3 (39.9 - 68.8)	32.3 (21.1 - 43.4)	30.7 (19.8 - 41.5)	33.8 (22.4 - 45.2)
2015	40.2 (27.7 - 52.6)	37.4 (25.4 - 49.3)	42.6 (29.8 - 55.4)	49.9 (36.1 - 63.8)	46.0 (32.7 - 59.3)	53.4 (39.1 - 67.7)	31.3 (20.4 - 42.3)	29.5 (18.9 - 40.2)	32.9 (21.7 - 44.2)
2016	39.1 (26.8 - 51.3)	36.9 (25.0 - 48.8)	41.1 (28.5 - 53.7)	48.4 (34.8 - 62.0)	45.3 (32.1 - 58.5)	51.2 (37.2 - 65.3)	30.6 (19.7 - 41.4)	29.2 (18.6 - 39.8)	31.8 (20.8 - 42.9)
2017	36.9 (25.0 - 48.8)	34.0 (22.6 - 45.4)	39.6 (27.2 - 51.9)	46.2 (32.9 - 59.6)	42.3 (29.6 - 55.0)	49.8 (35.9 - 63.6)	28.4 (17.9 - 38.8)	26.4 (16.3 - 36.4)	30.2 (19.4 - 41.0)
2018	36.1 (24.3 - 47.9)	33.9 (22.5 - 45.3)	38.1 (26.0 - 50.2)	45.3 (32.1 - 58.5)	42.4 (29.6 - 55.1)	48.0 (34.5 - 61.6)	27.5 (17.2 - 37.8)	26.1 (16.1 - 36.1)	28.8 (18.3 - 39.4)
2019	34.6 (23.0 - 46.1)	32.5 (21.3 - 43.6)	36.5 (24.7 - 48.4)	43.1 (30.2 - 55.9)	40.4 (27.9 - 52.8)	45.5 (32.3 - 58.7)	26.6 (16.5 - 36.7)	25.1 (15.3 - 34.9)	28.1 (17.7 - 38.5)

Supplementary Table S5. Absolute and relative changes of cause-specific avoidable mortality rate according to the type of areas from 1995 to 2019 in males[1]

Cause group	Absolute change (per 100,000 persons)						Relative change (%)					
	Preventable			Treatable			Preventable			Preventable		
	Total	Metro[2]	Non-metro[3]	Total	Metro[2]	Non-metro[3]	Total	Metro[2]	Non-metro[3]	Total	Metro[2]	Non-metro[3]
All avoidable causes of death	-276.8	-220.7	-315.1	-75.2	-70.9	-78.0	-68.0	-65.4	-68.8	-63.6	-63.7	-63.1
Infectious diseases	-7.2	-5.5	-8.3	-8.2	-6.9	-9.1	-80.0	-76.7	-81.2	-76.3	-76.2	-75.7
Tuberculosis	-7.6	-5.7	-8.9	-7.6	-5.7	-8.9	-92.4	-90.2	-93.4	-92.4	-90.2	-93.4
Others	0.4	0.2	0.5	-0.6	-1.1	-0.2	46.7	24.8	65.6	-24.3	-42.7	-9.1
Cancer	-91.9	-72.9	-103.2	0.1	-1.0	0.8	-65.9	-62.4	-67.0	1.3	-10.6	10.4
Stomach cancer[4]	-35.9	-29.3	-39.8	-	-	-	-81.9	-80.2	-82.3	-	-	-
Liver cancer[4]	-30.4	-22.8	-35.4	-	-	-	-66.9	-62.4	-68.4	-	-	-
Lung cancer[4]	-19.2	-16.6	-20.4	-	-	-	-50.6	-49.2	-50.3	-	-	-
Colorectal cancer[5]	-	-	-	0.1	-1.0	0.8	-	-	-	1.2	-11.8	11.4
Breast cancer[5,6]	-	-	-	-	-	-	-	-	-	-	-	-
Cervical cancer[6]	-	-	-	-	-	-	-	-	-	-	-	-
Others	-6.4	-4.3	-7.7	0.0	0.0	0.0	-52.4	-42.5	-57.2	2.0	1.2	3.2
Endocrine and metabolic diseases	-8.3	-8.1	-8.5	-8.5	-8.3	-8.7	-74.2	-75.3	-73.3	-74.3	-75.6	-73.4
Diabetes mellitus	-8.3	-8.1	-8.5	-8.3	-8.1	-8.5	-74.2	-75.3	-73.4	-74.2	-75.3	-73.4
Others[5]	-	-	-	-0.7	-0.4	-0.9	-	-	-	-49.8	-37.4	-56.2
Diseases of the nervous system (epilepsy)[5]	-	-	-	-0.7	-0.4	-0.9	-	-	-	-49.8	-37.4	-56.2
Diseases of the circulatory system	-47.5	-44.0	-50.0	-47.5	-44.0	-49.9	-77.9	-76.8	-78.6	-77.6	-76.5	-78.1
Ischaemic heart diseases	-3.6	-4.0	-3.6	-3.6	-4.0	-3.6	-38.9	-41.1	-38.3	-38.9	-41.1	-38.3
Cerebrovascular diseases	-35.4	-35.6	-35.4	-35.4	-35.6	-35.4	-84.3	-84.7	-84.0	-84.3	-84.7	-84.0
Others	-8.5	-4.5	-10.9	-8.5	-4.5	-10.9	-88.0	-78.5	-90.9	-85.5	-75.8	-88.2
Diseases of the respiratory system	-4.8	-6.3	-3.9	-6.1	-6.1	-5.7	-57.4	-69.8	-47.6	-41.6	-46.4	-36.4
Chronic lower respiratory diseases[4]	-3.8	-5.4	-2.8	-	-	-	-63.0	-74.3	-53.2	-	-	-
Pneumonia, not elsewhere classified[5]	-	-	-	3.0	1.9	4.0	-	-	-	66.5	43.5	84.9
Others	-1.1	-0.8	-1.1	-9.1	-8.0	-9.6	-43.6	-49.9	-37.6	-90.2	-92.5	-88.1
Diseases of the digestive system[5]	-	-	-	-2.6	-1.4	-3.3	-	-	-	-2.6	-1.4	-3.3
Gastric and duodenal ulcer	-	-	-	-2.1	-1.0	-2.7	-	-	-	-2.1	-1.0	-2.7
Others	-	-	-	-0.5	-0.4	-0.6	-	-	-	-0.5	-0.4	-0.6
Diseases of the genitourinary system[5]	-	-	-	-1.7	-3.2	-0.9	-	-	-	-35.9	-52.0	-21.5
Renal failure	-	-	-	-1.3	-2.8	-0.4	-	-	-	-29.9	-49.4	-11.6
Others	-	-	-	-0.4	-0.4	-0.5	-	-	-	-89.8	-90.4	-88.6
Diseases of pregnancy, childbirth, and perinatal period	0.0	0.0	0.0	0.8	0.9	0.7	0.0	0.0	0.0	193.3	317.1	129.5
Certain conditions originating in the perinatal period[5]	-	-	-	0.8	0.9	0.7	-	-	-	193.3	317.1	129.5
Others	0.0	0.0	0.0	0.0	0.0	0.0	0.0	0.0	0.0	0.0	0.0	0.0
Congenital malformations	0.0	0.0	0.0	-0.8	-0.6	-0.9	-45.2	-26.5	-100.0	-71.7	-64.2	-77.3
Congenital malformations of the circulatory system[5]	-	-	-	-0.8	-0.6	-0.9	-	-	-	-71.7	-64.2	-77.3
Others[4]	0.0	0.0	0.0	-	-	-	-45.2	-26.5	-100.0	-	-	-
Adverse effects of medical and surgical care[5]	-	-	-	0.0	0.1	-0.1	-	-	-	22.5	217.5	-31.3
Misadventures to patients during surgical and medical care	-	-	-	0.0	0.2	-0.1	-	-	-	38.1	695.8	-42.5
Others	-	-	-	0.0	0.0	0.0	-	-	-	-37.6	-100.0	42.8
Injuries[4]	-66.7	-49.7	-79.2	-	-	-	-58.7	-55.8	-59.3	-	-	-
Transport Accidents	-52.6	-35.6	-65.5	-	-	-	-87.1	-86.6	-86.9	-	-	-
Intentional self-harm	12.6	8.9	15.7	-	-	-	79.8	54.5	98.7	-	-	-
Others	-26.7	-23.0	-29.4	-	-	-	-71.3	-72.8	-69.7	-	-	-
Alcohol related and drug-related deaths[4]	-50.4	-34.1	-61.9	-	-	-	-78.0	-71.9	-80.4	-	-	-
Alcohol specific disorders and poisonings	-3.6	-1.9	-4.8	-	-	-	-24.9	-15.3	-29.8	-	-	-
Others	-46.7	-32.2	-57.1	-	-	-	-93.4	-92.2	-93.7	-	-	-

[1] Absolute change = mortality in 1995 – mortality in 2019; relative change (ratio) = (mortality in 1995 – mortality in 2019) / mortality in 1995 × 100; [2] metro: metropolitan area; [3] non-metro: non-metropolitan area; [4] only included in the category of preventable death [5] only included in the category of treatable death; [6] values for female subjects only

Supplementary Table S6. Absolute and relative changes of cause-specific avoidable mortality rate according to the type of areas from 1995 to 2019 in females[1]

Cause group	Absolute change (per 100,000 persons)						Relative change (%)					
	Preventable			Treatable			Preventable			Preventable		
	Total	Metro[2]	Non-metro[3]	Total	Metro[2]	Non-metro[3]	Total	Metro[2]	Non-metro[3]	Total	Metro[2]	Non-metro[3]
All avoidable causes of death	-89.6	-81.6	-94.8	-46.4	-47.6	-45.6	-68.0	-67.6	-67.6	-63.5	-65.4	-61.9
Infectious diseases	-1.6	-1.4	-1.7	-1.7	-1.8	-1.7	-74.8	-75.5	-73.7	-58.3	-64.3	-54.1
Tuberculosis	-1.6	-1.2	-1.8	-1.6	-1.2	-1.8	-92.4	-91.0	-93.0	-92.4	-91.0	-93.0
Others	0.0	-0.1	0.1	-0.1	-0.5	0.1	3.2	-25.0	28.0	-11.0	-38.4	12.5
Cancer	-25.1	-21.7	-27.2	-3.0	-4.0	-2.5	-64.6	-61.9	-65.8	-18.9	-23.3	-16.4
Stomach cancer[4]	-13.7	-11.3	-15.2	-	-	-	-80.6	-78.0	-81.7	-	-	-
Liver cancer[4]	-6.9	-6.1	-7.4	-	-	-	-67.6	-66.6	-67.7	-	-	-
Lung cancer[4]	-3.4	-3.1	-3.6	-	-	-	-39.4	-37.3	-40.9	-	-	-
Colorectal cancer[5]	-	-	-	-1.1	-1.6	-0.8	-	-	-	-22.1	-29.6	-16.0
Breast cancer[5][6]	-	-	-	1.9	1.5	2.0	-	-	-	41.4	29.0	48.5
Cervical cancer[6]	-0.5	-0.9	-0.2	-0.5	-0.9	-0.2	-33.7	-51.1	-14.5	-33.7	-51.1	-14.5
Others	-0.6	-0.4	-0.8	-3.3	-3.0	-3.5	-36.8	-25.0	-44.0	-67.3	-63.7	-70.3
Endocrine and metabolic diseases	-5.6	-6.4	-5.0	-5.9	-6.7	-5.4	-84.2	-88.5	-80.3	-84.0	-88.3	-80.1
Diabetes mellitus	-5.5	-6.4	-5.0	-5.5	-6.4	-5.0	-84.0	-88.4	-80.0	-84.0	-88.4	-80.0
Others[5]	-	-	-	-0.5	-0.2	-0.7	-	-	-	-57.7	-29.5	-69.3
Diseases of the nervous system (epilepsy)[5]	-	-	-	-0.5	-0.2	-0.7	-	-	-	-57.7	-29.5	-69.3
Diseases of the circulatory system	-30.0	-29.1	-30.5	-30.0	-29.1	-30.4	-86.3	-87.2	-85.4	-85.8	-86.7	-84.8
Ischaemic heart diseases	-2.2	-2.5	-2.0	-2.2	-2.5	-2.0	-64.7	-70.1	-59.9	-64.7	-70.1	-59.9
Cerebrovascular diseases	-22.6	-23.5	-21.9	-22.6	-23.5	-21.9	-88.0	-89.5	-86.6	-88.0	-89.5	-86.6
Others	-5.2	-3.0	-6.6	-5.2	-3.0	-6.5	-92.1	-87.4	-93.5	-88.6	-82.9	-90.2
Diseases of the respiratory system	-1.5	-2.0	-1.2	-2.9	-3.0	-2.8	-66.9	-75.2	-59.0	-50.6	-56.0	-46.3
Chronic lower respiratory diseases[4]	-1.5	-1.9	-1.3	-	-	-	-80.0	-83.5	-77.2	-	-	-
Pneumonia, not elsewhere classified[5]	-	-	-	0.6	0.1	0.9	-	-	-	32.1	4.0	51.1
Others	0.0	-0.1	0.1	-3.5	-3.1	-3.8	5.4	-23.7	29.1	-89.2	-90.7	-87.7
Diseases of the digestive system[5]	-	-	-	-1.0	-0.8	-1.1	-	-	-	-73.9	-75.0	-71.5
Gastric and duodenal ulcer	-	-	-	-0.6	-0.4	-0.7	-	-	-	-88.4	-83.1	-90.8
Others	-	-	-	-0.5	-0.4	-0.4	-	-	-	-61.1	-68.2	-54.2
Diseases of the genitourinary system[5]	-	-	-	-1.4	-2.0	-1.1	-	-	-	-47.3	-57.6	-38.5
Renal failure	-	-	-	-1.1	-1.7	-0.7	-	-	-	-41.0	-54.0	-29.3
Others	-	-	-	-0.4	-0.3	-0.4	-	-	-	-87.8	-89.0	-86.7
Diseases of pregnancy, childbirth, and perinatal period	0.0	0.0	0.0	0.7	0.7	0.7	0.0	0.0	0.0	232.7	252.5	218.6
Certain conditions originating in the perinatal period[5]	-	-	-	0.7	0.7	0.8	-	-	-	254.2	263.6	247.2
Others	0.0	0.0	0.0	0.0	0.0	0.0	0.0	0.0	0.0	-76.3	-100.0	-68.1
Congenital malformations	0.0	0.0	0.0	-0.7	-0.8	-0.7	276.1	73.7	484.3	-70.2	-81.0	-60.6
Congenital malformations of the circulatory system[5]	-	-	-	-0.7	-0.8	-0.7	-	-	-	-70.2	-81.0	-60.6
Others[4]	0.0	0.0	0.0	-	-	-	276.1	73.7	484.3	-	-	-
Adverse effects of medical and surgical care[5]	-	-	-	0.0	0.1	0.0	-	-	-	46.0	126.5	-10.2
Misadventures to patients during surgical and medical care	-	-	-	0.0	0.1	0.0	-	-	-	152.6	426.5	38.4
Others	-	-	-	0.0	0.0	0.0	-	-	-	-52.9	-51.8	-61.7
Injuries[4]	-19.9	-15.4	-23.0	-	-	-	-53.1	-48.9	-54.6	-	-	-
Transport Accidents	-17.3	-12.1	-21.0	-	-	-	-89.9	-90.2	-89.3	-	-	-
Intentional self-harm	6.3	6.1	6.4	-	-	-	98.8	99.0	95.1	-	-	-
Others	-8.9	-9.3	-8.5	-	-	-	-74.9	-78.5	-71.3	-	-	-
Alcohol related and drug-related deaths[4]	-6.0	-5.6	-6.2	-	-	-	-61.3	-62.3	-59.4	-	-	-
Alcohol specific disorders and poisonings	1.4	1.3	1.6	-	-	-	217.7	229.3	218.6	-	-	-
Others	-7.4	-6.9	-7.8	-	-	-	-81.3	-81.8	-80.6	-	-	-

[1] Absolute change = mortality in 1995 – mortality in 2019; relative change (ratio) = (mortality in 1995 – mortality in 2019) / mortality in 1995 × 100; [2] metro: metropolitan area; [3] non-metro: non-metropolitan area; [4] only included in the category of preventable death [5] only included in the category of treatable death; [6] values for female subjects only

Supplementary Table S7. Absolute and relative differences of all-cause and avoidable mortality between metropolitan and non-metropolitan areas from 1995 to 2019

Year	All-cause death						Avoidable death					
	Absolute difference			Relative difference			Absolute difference			Relative difference		
	Total	Male	Female	Total	Male	Female	Total	Male	Female	Total	Male	Female
1995	132.00	236.90	54.80	1.20	1.27	1.11	72.66	132.75	20.45	1.24	1.30	1.11
1996	117.24	208.67	49.21	1.18	1.24	1.10	71.77	128.15	23.84	1.25	1.30	1.13
1997	121.24	216.17	52.02	1.19	1.26	1.11	69.90	127.14	21.53	1.24	1.31	1.12
1998	106.38	193.35	43.32	1.17	1.23	1.09	64.79	118.80	19.64	1.24	1.30	1.12
1999	110.35	191.62	52.15	1.18	1.24	1.12	62.01	109.30	22.37	1.23	1.28	1.14
2000	94.13	174.76	39.05	1.16	1.22	1.09	55.05	101.42	17.63	1.21	1.27	1.11
2001	86.27	155.07	38.54	1.15	1.21	1.09	52.12	93.14	19.91	1.21	1.25	1.13
2002	83.60	151.84	38.06	1.15	1.21	1.09	53.67	95.43	20.67	1.22	1.27	1.14
2003	87.08	149.87	43.97	1.17	1.22	1.11	52.75	92.00	21.51	1.22	1.27	1.15
2004	76.63	137.64	34.73	1.15	1.21	1.09	47.89	86.12	17.49	1.21	1.26	1.13
2005	68.05	116.70	34.77	1.14	1.18	1.10	41.27	73.55	15.86	1.19	1.24	1.12
2006	65.65	115.98	32.15	1.14	1.19	1.09	40.55	70.60	16.57	1.20	1.24	1.14
2007	65.30	110.43	33.47	1.15	1.18	1.10	43.50	75.45	17.21	1.23	1.28	1.15
2008	70.11	120.04	35.21	1.17	1.21	1.11	43.18	73.89	17.40	1.25	1.29	1.16
2009	63.62	105.76	34.60	1.16	1.19	1.12	39.53	65.61	17.45	1.23	1.27	1.17
2010	57.93	101.90	27.91	1.14	1.19	1.10	35.34	60.39	13.87	1.21	1.25	1.14
2011	54.55	95.81	26.63	1.14	1.18	1.10	34.30	58.30	13.58	1.22	1.26	1.15
2012	52.58	89.73	27.46	1.14	1.17	1.10	31.37	51.73	13.61	1.21	1.24	1.16
2013	45.19	81.79	20.94	1.12	1.17	1.08	26.00	43.15	10.85	1.18	1.21	1.13
2014	43.28	78.49	19.89	1.12	1.17	1.08	26.12	42.07	11.58	1.19	1.21	1.15
2015	47.29	80.57	24.80	1.14	1.18	1.10	26.40	41.55	12.22	1.20	1.22	1.16
2016	41.42	74.51	18.33	1.13	1.17	1.08	24.29	38.58	10.22	1.20	1.22	1.14
2017	45.06	73.67	24.47	1.14	1.18	1.11	22.56	34.42	10.31	1.19	1.20	1.16
2018	42.36	68.28	23.74	1.13	1.16	1.10	22.58	33.03	11.30	1.20	1.20	1.17
2019	42.45	66.92	23.95	1.14	1.17	1.11	20.83	31.39	9.25	1.19	1.20	1.14

Supplementary Table S8. Absolute and relative differences of preventable and treatable mortality between metropolitan and non-metropolitan areas from 1995 to 2019

Year	Preventable death						Treatable death					
	Absolute difference			Relative difference			Absolute difference			Relative difference		
	Total	Male	Female	Total	Male	Female	Total	Male	Female	Total	Male	Female
1995	66.44	120.59	19.41	1.30	1.36	1.16	6.22	12.16	1.04	1.07	1.11	1.01
1996	64.27	113.41	21.87	1.31	1.35	1.20	7.50	14.74	1.97	1.09	1.15	1.03
1997	62.21	110.64	20.83	1.30	1.35	1.19	7.69	16.49	0.70	1.09	1.16	1.01
1998	58.26	104.83	18.98	1.30	1.35	1.18	6.53	13.97	0.66	1.08	1.14	1.01
1999	54.96	96.68	19.84	1.30	1.34	1.20	7.05	12.61	2.54	1.09	1.12	1.04
2000	49.05	88.37	16.87	1.27	1.31	1.17	6.00	13.05	0.76	1.07	1.13	1.01
2001	46.89	83.56	17.83	1.27	1.31	1.20	5.23	9.58	2.08	1.07	1.10	1.03
2002	48.42	86.10	18.53	1.28	1.33	1.21	5.25	9.33	2.14	1.07	1.10	1.03
2003	48.08	83.38	19.90	1.29	1.32	1.23	4.67	8.63	1.61	1.07	1.10	1.03
2004	43.14	78.14	15.11	1.27	1.32	1.18	4.76	7.98	2.38	1.07	1.10	1.04
2005	36.11	65.25	13.08	1.24	1.28	1.16	5.16	8.30	2.78	1.08	1.11	1.06
2006	35.65	62.94	13.64	1.25	1.29	1.19	4.90	7.67	2.92	1.08	1.11	1.06
2007	37.38	65.71	13.80	1.28	1.32	1.20	6.12	9.73	3.41	1.11	1.15	1.08
2008	35.94	62.51	13.40	1.28	1.33	1.20	7.24	11.38	4.00	1.15	1.19	1.10
2009	32.86	55.44	13.54	1.26	1.29	1.21	6.66	10.16	3.92	1.14	1.18	1.11
2010	29.78	51.33	11.19	1.25	1.28	1.18	5.56	9.06	2.69	1.12	1.16	1.07
2011	28.32	48.17	10.92	1.25	1.28	1.19	5.98	10.13	2.66	1.14	1.19	1.08
2012	25.27	43.35	9.19	1.23	1.26	1.17	6.10	8.37	4.41	1.15	1.16	1.14
2013	21.43	36.29	8.01	1.21	1.23	1.16	4.57	6.86	2.84	1.11	1.14	1.09
2014	21.22	34.92	8.46	1.22	1.23	1.18	4.90	7.15	3.12	1.13	1.15	1.10
2015	21.14	34.10	8.78	1.23	1.24	1.19	5.26	7.45	3.44	1.14	1.16	1.12
2016	20.06	32.65	7.55	1.23	1.24	1.17	4.23	5.92	2.68	1.11	1.13	1.09
2017	16.98	26.96	6.48	1.21	1.21	1.16	5.58	7.47	3.83	1.16	1.18	1.15
2018	18.38	27.36	8.57	1.23	1.22	1.22	4.19	5.67	2.72	1.12	1.13	1.10
2019	16.76	26.26	6.29	1.22	1.22	1.16	4.08	5.13	2.96	1.13	1.13	1.12
